# Ethiopian women's tokophobia of childbirth and its predictors: a systematic review and meta-analysis

**DOI:** 10.3389/fgwh.2024.1334103

**Published:** 2024-08-02

**Authors:** Nega Tezera Assimamaw, Almaz Tefera Gonete, Dessie Abebaw Angaw, Masresha Asmare Techane, Mengistu Mekonnen Kelkay, Bewuketu Terefe

**Affiliations:** ^1^ Department of Pediatrics and Child Health Nursing, School of Nursing, College of Medicine and Health Sciences, University of Gondar, Gondar, Ethiopia; ^2^ Department of Epidemiology and Biostatistics, Institute of Public Health, College of Medicine and Health Sciences, University of Gondar, Gondar, Ethiopia; ^3^ Community Health Nursing, School of Nursing, College of Medicine and Health Sciences, University of Gondar, Gondar, Ethiopia

**Keywords:** fear of childbirth, pregnant mothers, Ethiopia, systematic review and meta-analysis, prevelance

## Abstract

**Background:**

Fear of childbirth is recognized as a growing problem in developing countries, including Ethiopia. The impact of this fear on women's reproductive choices and decisions is significant. Therefore, the systematic review and meta-analysis will help to consolidate the existing research on childbirth-related fear in Ethiopia. Synthesizing the findings and providing a pooled prevalence estimate, can contribute to a better understanding of the scale of the problem in the country.

**Objective:**

This systematic review and meta-analysis assessed the pooled prevalence of childbirth-related fear and its associated factors among pregnant mothers in Ethiopia.

**Methods:**

PubMed, Google Scholar, and African Journals Online were searched for included articles. A weighted inverse-variance random-effects model was used to estimate the prevalence of childbirth-related fear. Variations in the pooled estimates of the prevalence were adjusted through subgroup analysis according to the specific region where the study was conducted. Funnel plot and Egger's regression test were used to check for publication bias. STATA version 14 statistical software was used for meta-analysis.

**Results:**

A total of 2,015 pregnant mothers were included. The combined prevalence of fear of childbirth among pregnant mothers was found to be 21% (95% CI: 19–22; *I*^2^ = 0.00%, *p* value < 0.001). Based on the subgroup analysis, the prevalence of fear of childbirth among pregnant mothers was 24% in SNNPRs, 25% in Oromia, and 11% in Addis Ababa.

**Conclusion:**

The findings of the meta-analysis indicating a high prevalence of fear of childbirth among pregnant mothers in Ethiopia and identifying associated risk factors highlight the importance of addressing this issue within the healthcare system. Integrating prevention-based services for mothers with childbirth fears into the antenatal care model could be a valuable approach to support women and mitigate the impact of fear on their reproductive experiences.

**Systematic Review Registration:**

https://www.crd.york.ac.uk/prospero/#myprospero, identifier [CRD42023411103].

## 1 Introduction

Tokophobia refers to an intense fear or phobia of childbirth. It can manifest as severe anxiety, panic attacks, or avoidance behaviors related to pregnancy and childbirth. While tokophobia can affect individuals of any gender, it is more commonly reported in women (1–5).

The reported of 6%–10% in pregnant women reflects a general estimate of fear of childbirth (FOC) prevalence worldwide (6). However, the magnitude of tokophobia or FOC in Africa as a whole is not well documented due to limited research specifically focusing on this topic across the continent. However, some studies have explored the fear of childbirth in individual African countries, providing insights into the prevalence within those specific contexts. For example, a study conducted in Malawi reported a prevalence of fear of childbirth of 20% among pregnant women. Another study in Kenya found a prevalence of 58.6% among pregnant women. These studies highlight that the fear of childbirth is a significant concern in these specific regions (7, 8).

Several factors can contribute to extreme fear of childbirth among women, including cultural and societal factors. Cultural beliefs, traditions, and societal expectations around childbirth can play a significant role in shaping women's fears. In some cultures, childbirth is considered a challenging and potentially dangerous event that can lead to heightened anxiety (9–11). Limited access to accurate and comprehensive information about pregnancy, childbirth, and available medical interventions can contribute to fear. When women lack knowledge about the birthing process and potential complications, they may feel anxious and fearful (12–14). Women who have had previous negative or traumatic experiences during childbirth, such as complications, emergency interventions, or loss of a child, may develop a heightened fear of subsequent pregnancies and deliveries (2, 15–18). Insufficient access to quality maternity care, including prenatal education, antenatal visits, and emotional support, can contribute to fear and anxiety. When women do not have a trusted healthcare provider or a supportive network during pregnancy and childbirth, their fears may intensify (19–21).

Addressing extreme fear of childbirth requires a comprehensive approach that considers various individual and systemic factors. It is important to recognize that the fear of childbirth is a complex issue influenced by a range of factors at different levels.

At the individual level, providing education and information about the birthing process, addressing misconceptions and anxieties, and offering psychological support through counseling or therapy can be beneficial. This can help women understand the physiological and psychological aspects of childbirth, provide coping mechanisms, and empower them to make informed choices (21–25). Additionally, healthcare providers can offer personalized care and build trusting relationships with pregnant women, creating a safe and supportive environment during pregnancy, labor, and delivery. This can help alleviate fears and enhance the overall childbirth experience. At the systemic level, there are several considerations. It is essential to ensure that healthcare systems have adequate resources, including skilled healthcare providers, accessible and high-quality antenatal and maternity care services, and appropriate medical interventions when needed. This includes promoting evidence-based practices, respectful maternity care, and continuity of care throughout the perinatal period (26–28).

Furthermore, addressing the societal and cultural factors that contribute to the fear of childbirth is crucial. This may involve challenging negative cultural beliefs and promoting positive narratives about childbirth. Engaging communities, religious leaders, and other stakeholders in discussions and awareness campaigns can help reduce stigma and create a supportive social environment for pregnant women (29–31).

Efforts are being made in Ethiopia and globally to address the fear of childbirth and improve maternal healthcare services. These include initiatives to increase access to quality prenatal care, provide education and information about childbirth, promote supportive and respectful maternity care, and enhance mental health support for pregnant women.

It is important to note that the specific predictors and prevalence of tokophobia among Ethiopian women require a systematic review and meta-analysis of existing studies conducted in Ethiopia. Such studies would provide a more comprehensive understanding of the factors influencing tokophobia in this specific population. Thus, healthcare providers and policymakers can use this information to develop targeted interventions and support systems for women during the perinatal period.

## 2 Methods and materials

### 2.1 Reporting

This review was undertaken in accordance with the Preferred Reporting Items for Systematic Reviews and Meta-Analyses (PRISMA guideline) (32) and was registered on the International Prospective Registered of Systematic Review [PROSPERO 2023 (CRD42023411103)] accessed at https://www.crd.york.ac.uk/prospero/#myprospero.

### 2.2 Information source and search strategy

To develop robust literature search strategies, we used the “Population, Intervention, Comparator, Outcomes (PICO)” framework. Based on a preliminary assessment of the appropriate Medical Subject Headings (MeSH terms), keywords, and synonyms, a search strategy was developed.

Identify the main concepts: pregnant mothers who had to be followed up in health institutions or facilities and living in Ethiopia (P), investigation on tokophobia of childbirth among pregnant women (I), pregnant women without tokophobia (C), and tokophobia of childbirth (O).

Medical Subject Headings (MeSH terms): MeSH terms are standardized terms used to index articles in PubMed and other biomedical databases. By searching multiple electronic databases, such as PubMed and Google Scholar, as well as African Journals Online, we accessed several scholarly articles. Furthermore, including a manual search of the bibliographies of relevant articles can help identify additional studies that may not have been captured in the initial database search. Searching for gray literature on university websites and online repositories is also a valuable step to ensure a comprehensive review of available evidence. Thus, the MeSH terms are “Pregnant mothers”, “Predictors”, “Prevalence”, “tokophobia”, and “Ethiopia”.

Keywords and synonyms: In addition to MeSH terms, identify relevant keywords and synonyms for each concept. These can be specific terms, phrases, or abbreviations related to the research question. This includes “pregnant women”, and “risk factors” OR “determinate” OR “contributing factors” OR “associated factors”, and “burden” OR “magnitude” OR “incidence” OR “epidemiology”, “Fear of childbirth”, “childbirth anxiety” OR “tokophobia of pregnancy” OR “fear of pregnancy”.

Combine search terms: Combine MeSH terms, keywords, and synonyms using Boolean operators such as AND and OR to create search strings. “Pregnant mothers” [MeSH Terms] OR “pregnant women” AND “Predictors” [MeSH Terms] OR “risk factors” OR “determinate” OR “contributing factors” OR “associated factors” AND “Prevalence” [MeSH Terms] OR “burden” OR “magnitude” OR “incidence” OR “epidemiology” AND “tokophobia of childbirth” [MeSH Terms] OR “tokophobia of childbirth” OR “childbirth anxiety” OR “fear of pregnancy” OR “fear of pregnancy” AND “Ethiopia” [MeSH Term].

In due course, evaluate the relevance and comprehensiveness of the retrieved articles. Adjust and refine the search strategy as needed by adding or removing terms to improve the precision and recall of the search results.

### 2.3 Study inclusion criteria

To ensure the inclusion of relevant studies, it is important to establish clear criteria for study selection in our systematic review. These criteria help determine which studies are eligible for inclusion based on specific characteristics and research questions.

Study design: Cross-sectional, cohort, and case-control studies were included in the scope of the search.

Geographical location: studies conducted in Ethiopia

Population: pregnant women in Ethiopia.

Language and Publication Status: Only English language articles were included in the review. All published data, including peer-reviewed articles, were considered. Gray literature, which includes theses was also included in the review.

Date of publication: we considered all studies in our systematic review.

### 2.4 Exclusion and inclusion criteria

The following criteria were considered:

Exclusion of Studies: Studies conducted on topics other than fear of childbirth among pregnant mothers were excluded. This ensures that the focus of our review remains specific to the research question at hand.

Exclusion of Editorial Comments: Editorial comments or opinion pieces were excluded from the review. This helps maintain the rigor and objectivity of the included studies.

Exclusion of Conference Proceedings: Studies published only as conference proceedings were excluded. This decision ensures that the included studies have undergone a peer-review process and are more likely to meet the required standards of quality and rigor.

Exclusion of Qualitatively Described Works: Qualitatively described works that may lack quantitative data or specific measurement of fear of childbirth were excluded. This helps ensure that the included studies provide relevant and measurable information on the fear of childbirth and associated factors.

Exclusion of Unpublished or Non-English Studies: Studies that have not been published or translated into English were excluded. This decision ensures that the included studies are accessible to the readers and can be appropriately assessed for quality and relevance.

Full Text Availability: Studies for which the full text was not available were excluded. This criterion helps ensure that you have access to the complete information necessary for a comprehensive evaluation and analysis.

Inclusion of Prevalence and/or Related Factors: Studies reporting on the prevalence of fear of childbirth and/or at least one related factor were included. This criterion ensures that the included studies provide relevant data on the prevalence and associated factors of fear of childbirth among pregnant mothers in Ethiopia.

### 2.5 Quality assessment tool

We used the Joanna Briggs Institute Meta-Analysis of Statistics Assessment and Review Tool (JBI-MAStARI) (33) for the critical appraisal of the included studies in our systematic review. The JBI critical appraisal checklist for cross-sectional studies, consisting of nine criteria, was used to assess the quality of the included studies. Each criterion was scored as either “Yes” (1) or “No” (0). The nine criteria of the JBI critical appraisal checklist for cross-sectional studies are as follows: (1) Was the sample frame appropriate to address the target population? (2) Were study participants sampled in an appropriate manner? (3) Was the sample size adequate? (4) Were the study subjects and the setting described in detail? (5) Was the data analysis conducted with sufficient coverage of the identified sample? (6) Was the response rate adequate, and if not, was the low response rate managed appropriately? (7) Were valid methods used for the identification of the condition? (8) Was the condition measured in a standard, reliable way for all participants? (9) Was the response rate adequate, and if not, was the low response rate managed appropriately? The total score for each study ranged from 0 to 9, with a higher score indicating better quality. Studies that scored 50% and above (i.e., 5 or more out of 9) on the quality assessment criteria were considered high quality and had a low risk of bias. It is important to have two independent appraisers (NT, BT) evaluate the study quality to enhance the reliability and validity of the assessment. Any disagreements between appraisers can be resolved through discussion and consensus.

### 2.6 Data extraction tool and process

Two authors, N.T and BT, independently reviewed the abstracts and full-text articles for data extraction in our systematic review. The specific information that was extracted included the following: Name of the First Author: The name of the primary author of each study was recorded. Country where Research was Conducted: The country where each study was conducted and documented. Study Design: The study design employed in each included study was recorded. Study Setting: The setting in which the study took place was documented. Year of Study: The year in which each study was conducted and recorded. Sample Size: The sample size of each study was extracted. In cases of disagreement between the two authors during the data extraction process, it is good practice to resolve the disagreement through discussion and consensus.

### 2.7 Measurement of the outcome

Based on the included studies' description, the Wijma Delivery Expectation/Experience Questionnaire (W-DEQ) was used to measure childbirth fear severity. The W-DEQ scores were categorized into different degrees of fear as follows: Low degree fear: W-DEQ sum of 38 or below. Moderate degree of fear: W-DEQ sum between 38 and 65.9. High degree of fear: W-DEQ sum between 66 and 84.9. Severe degree fear: W-DEQ sum of 85 or above (34). Using this categorization, we were able to assess the prevalence of fear of childbirth among pregnant women. Prevalence was determined by dividing the number of pregnant women most probably feared at the moment (based on their W-DEQ scores falling within the specific fear categories) by the total number of participants in the study.

### 2.8 Statistical analysis

A weighted inverse-variance random-effects model was used to estimate the prevalence of fear of childbirth. This model considers the variability within and between studies, assigning more weight to studies with larger sample sizes and smaller variances. The random-effects model which accounts for potential heterogeneity across studies (35) was used to estimate the prevalence of fear of childbirth. Subgroup analysis was conducted based on the region where the included studies were conducted. This analysis helps to explore potential sources of heterogeneity and assess whether the prevalence of fear of childbirth differs across different regions. Heterogeneity across studies was assessed using the *I*^2^ statistic. Heterogeneity was considered low, moderate, and high when the *I*^2^ values were approximately 25%, 50%, and 75%, respectively. The *I*^2^ statistic quantifies the proportion of total variation in estimates due to heterogeneity (36). Funnel plot and Egger's regression test were used to check publication bias and trim and fill analysis was used to adjust for bias. Funnel plots and Egger's regression tests were used to assess publication bias. Funnel plots visualize the distribution of study effect sizes against their precision, and Egger's regression test evaluates the asymmetry of the funnel plot. If publication bias was identified, trim and fill analysis was used to adjust for it (37). STATA version 14 statistical software was used for conducting the meta-analysis. STATA is a widely used statistical software package that provides various tools and functions for analyzing and synthesizing data in meta-analyses. Sensitivity analysis was performed to assess the impact of each individual study on the overall estimate. This analysis helps evaluate the robustness and stability of the findings by examining how the results change when each study is excluded separately.

## 3 Results

### 3.1 Search results and study characteristics

**Figure 1:**
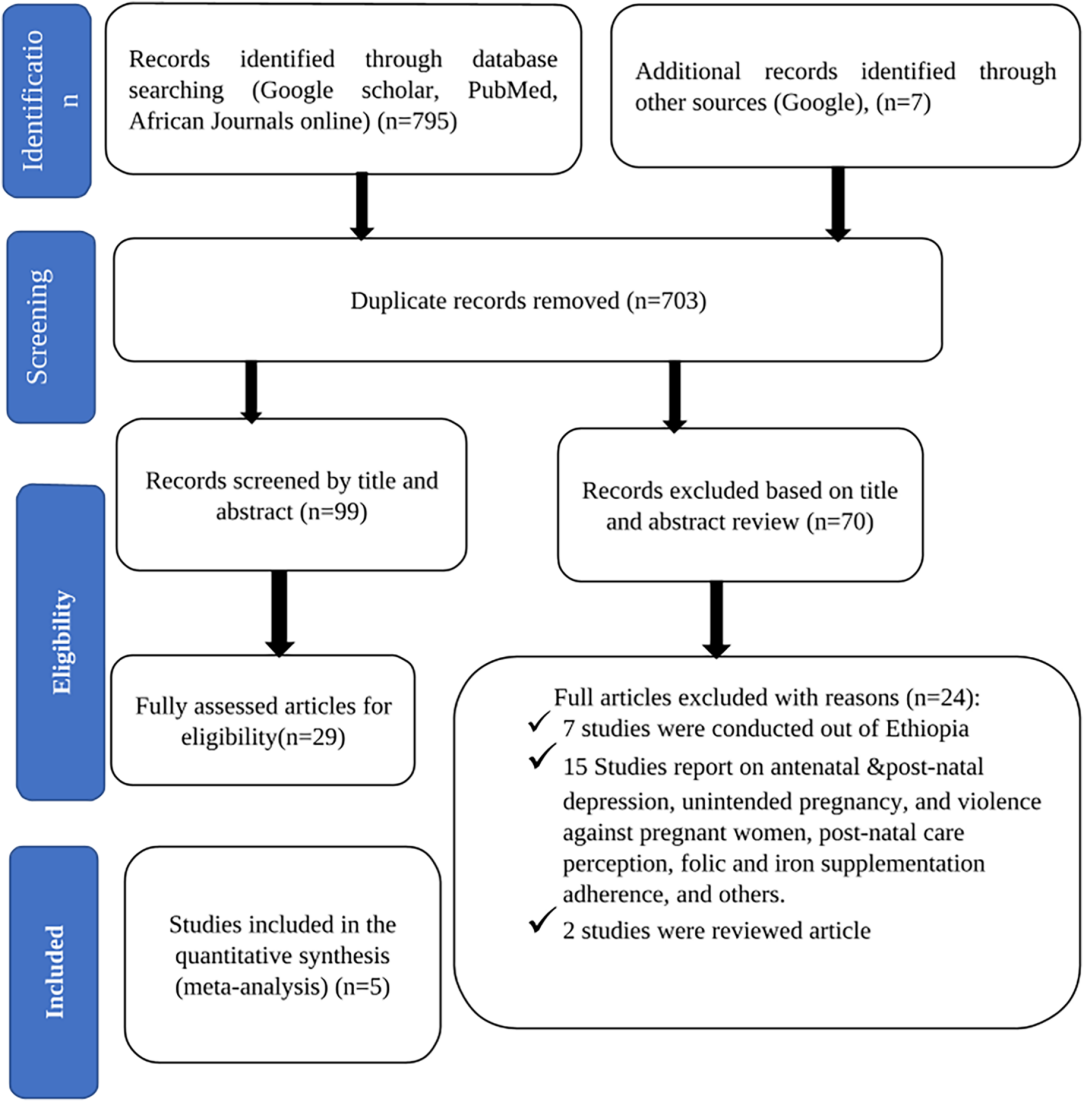
PRISMA flow diagram of identification and selection of studies for this systematic review and meta-analysis.

Eight hundred and two original article records were found through the online search from PubMed, Google Scholar, and African Journals online (AJO), of which 703 duplicate records were removed. Of the 99 articles screened for titles and abstracts, 70 articles were excluded as irrelevant. A total of 29 articles were reviewed in full text. In addition, 24 articles were excluded on the basis of predetermined eligibility criteria. Research studies have focused on antenatal and postnatal depression, unintended pregnancy, violence against pregnant women, post-natal care perceptions, and folic and iron supplementation adherence, among other topics. Finally, 5 articles were included in the meta-analysis (Figure 1).

### 3.2 Characteristics of the included studies

Five studies were conducted in Ethiopia between 2018 and 2022. Of these, two were in Southern Nations, Nationalities, and People's Region (SNNPs) (38, 39), two in Oromia (40, 41), and one in Addis Abeba (42). All studies accessed through the search were cross-sectional. Sample sizes ranged from 304 (41) to 482 (40) (Table 1).

**Table 1:** Characteristics of systemic review and meta-analysis articles (*n* = 5).

Author/year	Study area	Study Design	Sample size	Prevalence of fear of childbirth (95% CI)	Quality
Aynalem Y, et al./2018	SNNPRs	Facility-based cross-sectional study	423	24.2	Low risk
Robera DB, et al./2022	Oromia	Facility-based cross-sectional study	304	28.9	Low risk
Tiruset G, et al./2020	SNNPRs	Institution-based cross-sectional study	401	24.5	Low risk
Abebe T/2021	Addis Ababa	Institution-based cross-sectional study	405	11.3	Low risk
Adisalem D, et al./2022	Oromia	Community-based cross-sectional study	482	23.3	Low risk

### 3.3 Quality of the included studies

In all cross-sectional analyses, no methodological defects or significant bias were detected using the JBI critical appraisal checklist (Table 2).

**Table 2:** The Joanna Briggs Institute (JBI) prevalence critical appraisal tool.

S/N	Criteria	Yes	No	Unclear	Not applicable
1.	Was the sample frame appropriate to address the target population?	✓			
2.	Were study participants sampled in an appropriate way?	✓			
3.	Was the sample size adequate?	✓			
4.	Were the study subjects and the setting described in detail?	✓			
5.	Was the data analysis conducted with sufficient coverage of the identified sample?	✓			
6.	Were valid methods used for the identification of the condition?	✓			
7.	Was the condition measured in a standard, reliable way for all participants?	✓			
8.	Was there an appropriate statistical analysis?	✓			
9.	Was the response rate adequate, and if not, was the low response rate managed appropriately?	✓			

### 3.4 Meta-analysis

#### 3.4.1 Prevalence of fear of childbirth among pregnant mothers in Ethiopia

**Figure 2:**
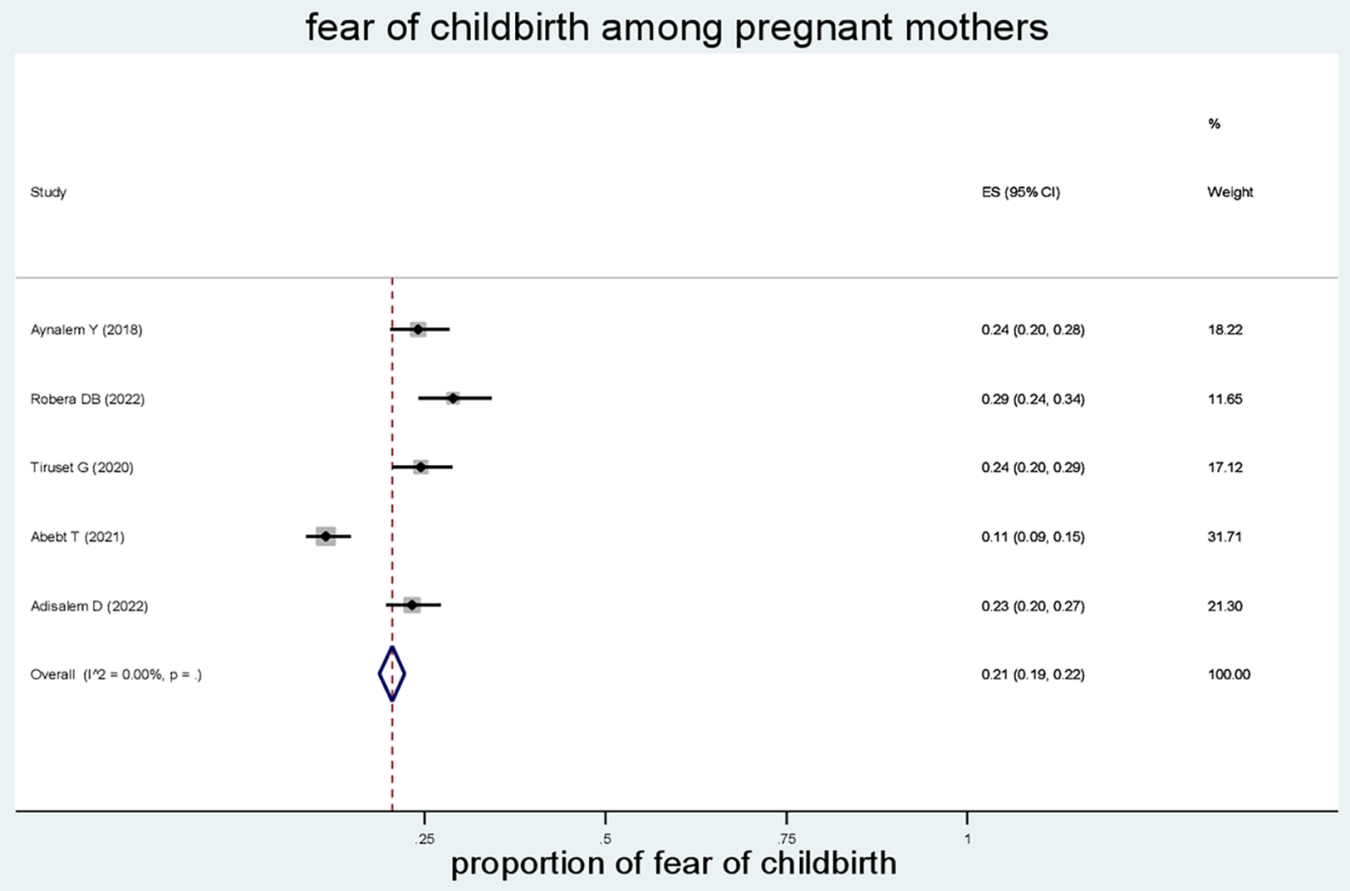
Forest plot of the pooled estimates (ES) of fear of childbirth among pregnant mothers. Each segment’s midpoint and length indicated prevalence and a 95% CI, whereas the diamond shape showed the combined prevalence of all studies.

The combined prevalence of fear of childbirth among pregnant mothers was found to be 21% (95% CI: 19–22; *I*^2^ = 0.00%, *P* value <0.001) (Figure 2).

#### 3.4.2 Subgroup analysis

**Figure 3:**
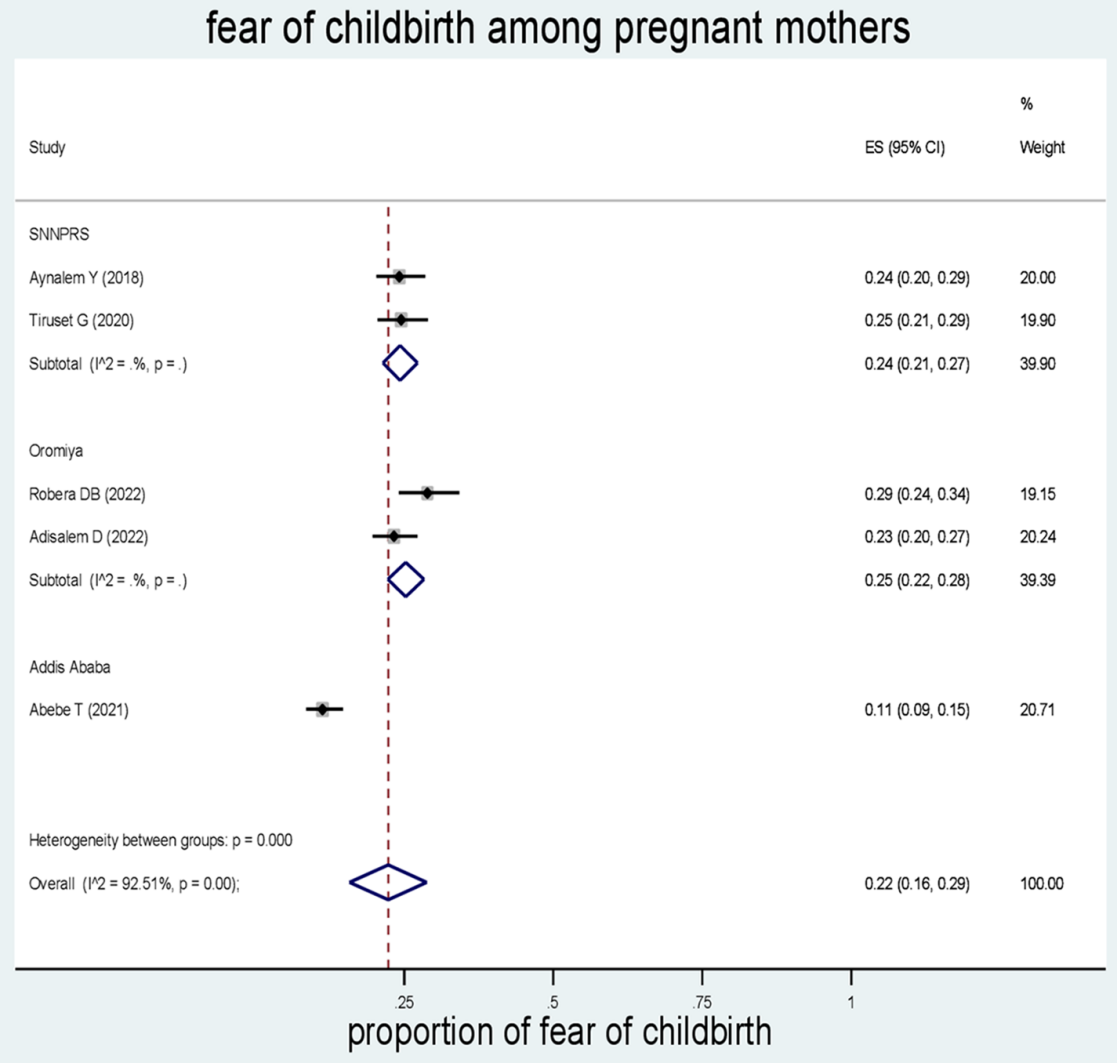
Forest plot of the pooled estimates (ES) of fear of childbirth among pregnant mothers. The midpoint and the length of each segment indicated prevalence and a 95% CI, whereas the diamond shape showed the combined prevalence of the studies.

Subgroup analysis based on the region, where the studies were conducted. The results revealed that the prevalence of fear of childbirth among pregnant mothers was 24% in SNNPRs, 25% in Oromia, and 11% in Addis Ababa (Figure 3).

#### 3.4.3 Sensitivity analysis

The sensitivity analysis showed that there was little change that could not affect the overall outcome estimate too much (Table 3).

**Table 3:** Sensitivity analysis of the prevalence of fear of childbirth among pregnant women in Ethiopia.

Study omitted	Estimate	95% confidence interval (CI)
Aynalem Y (2018)	15.40	11.67, 19.13
Robera DB (2022)	15.26	11.58, 18.94
Tiruset G (2020)	15.39	11.67, 19.12
Abebe T (2021)	24.85	18.92, 30.78
Adisalem D (2022)	15.35	11.58, 19.11

#### 3.4.4 Publication bias

**Figure 4:**
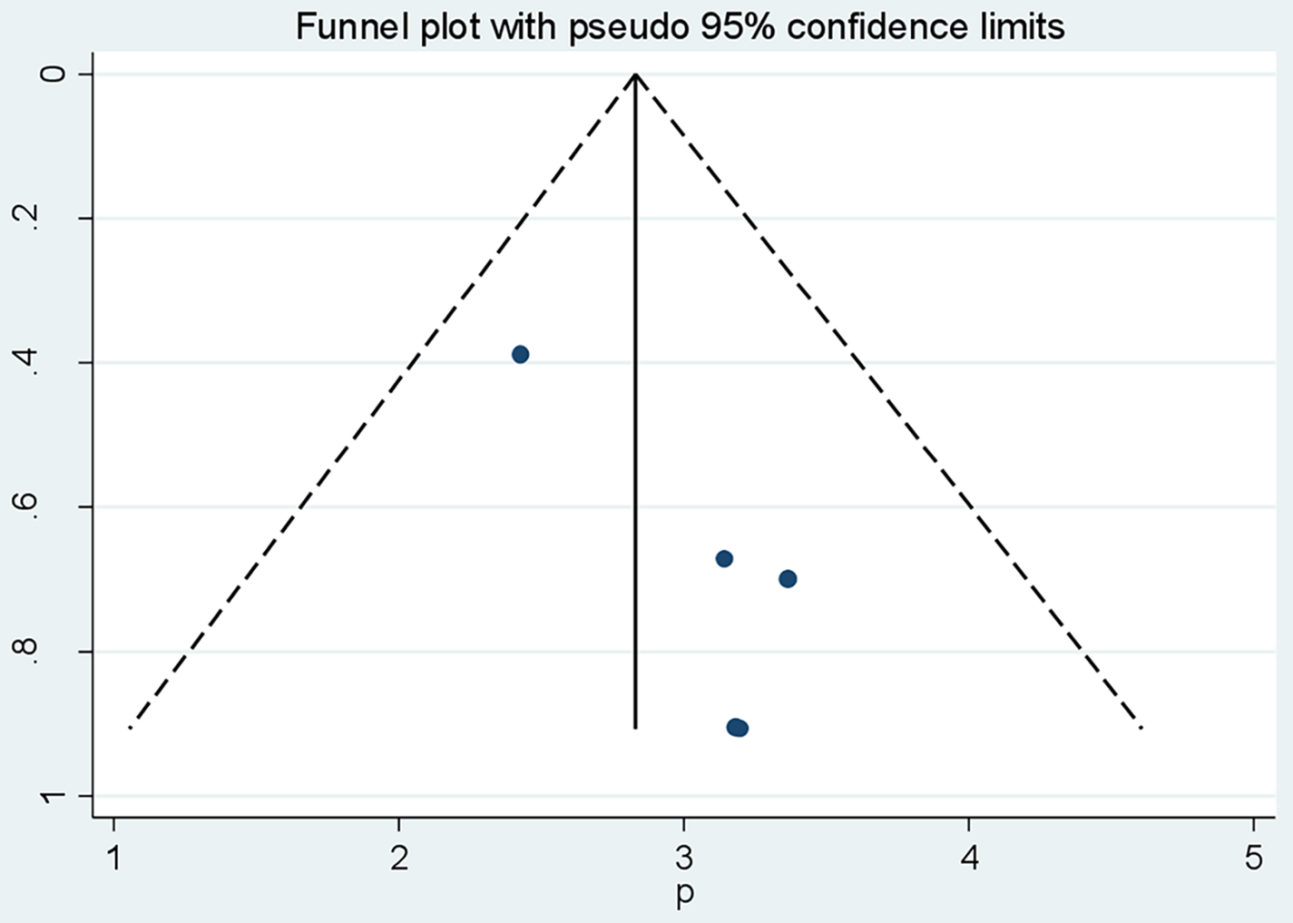
Funnel plot vertical lines estimate the effect size, whereas a diagonal line measures the precision of individual studies with a 95% confidence limit.

A funnel plot was employed to observe the symmetry of publications (Figure 4), and Egger's test showed no publication bias (*P* value = 0.604).

#### 3.4.5 Test for funnel plot asymmetry (egger's test)

Test of H0: no small-study effects *P* = 0.022.

The estimated bias coefficient (intercept) is 18.63 with a standard error of 4.23, giving a *p*-value of 0.02.

**Figure 5:**
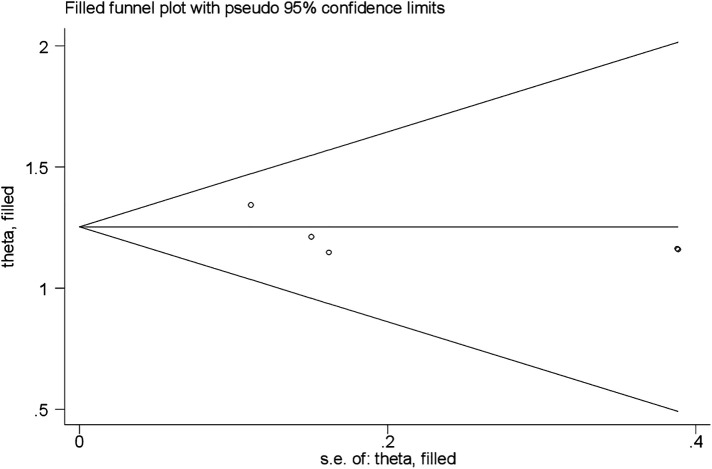
Funnel plot fill and trim analysis of fear of childbirth among pregnant mothers in Ethiopia.

There was a statistical publication bias as measured by Egger tests with a *p* = 0.02. Furthermore, a filled funnel trim analysis was conducted to further investigate publication bias, but no studies were found to be significantly biased (Figure 5; Table 4).

**Table 4:** Test for funnel plot asymmetry (egger's test).

Number of studies = 5	Root MSE = 1.533					
Std_Eff	Coef.	Std. Err.	*t*	*P* > *t*	[95% conf. interval]	
Slope	−.1601433	.0840882	−1.90	0.153	−.4277496	.107463
Bias	18.63886	4.235586	4.40	0.022	5.159339	32.11839

### 3.5 Determinant factors associated with the fear of childbirth among pregnant mothers

#### 3.5.1 Pregnancy and obstetrics-related complications

**Figure 6:**
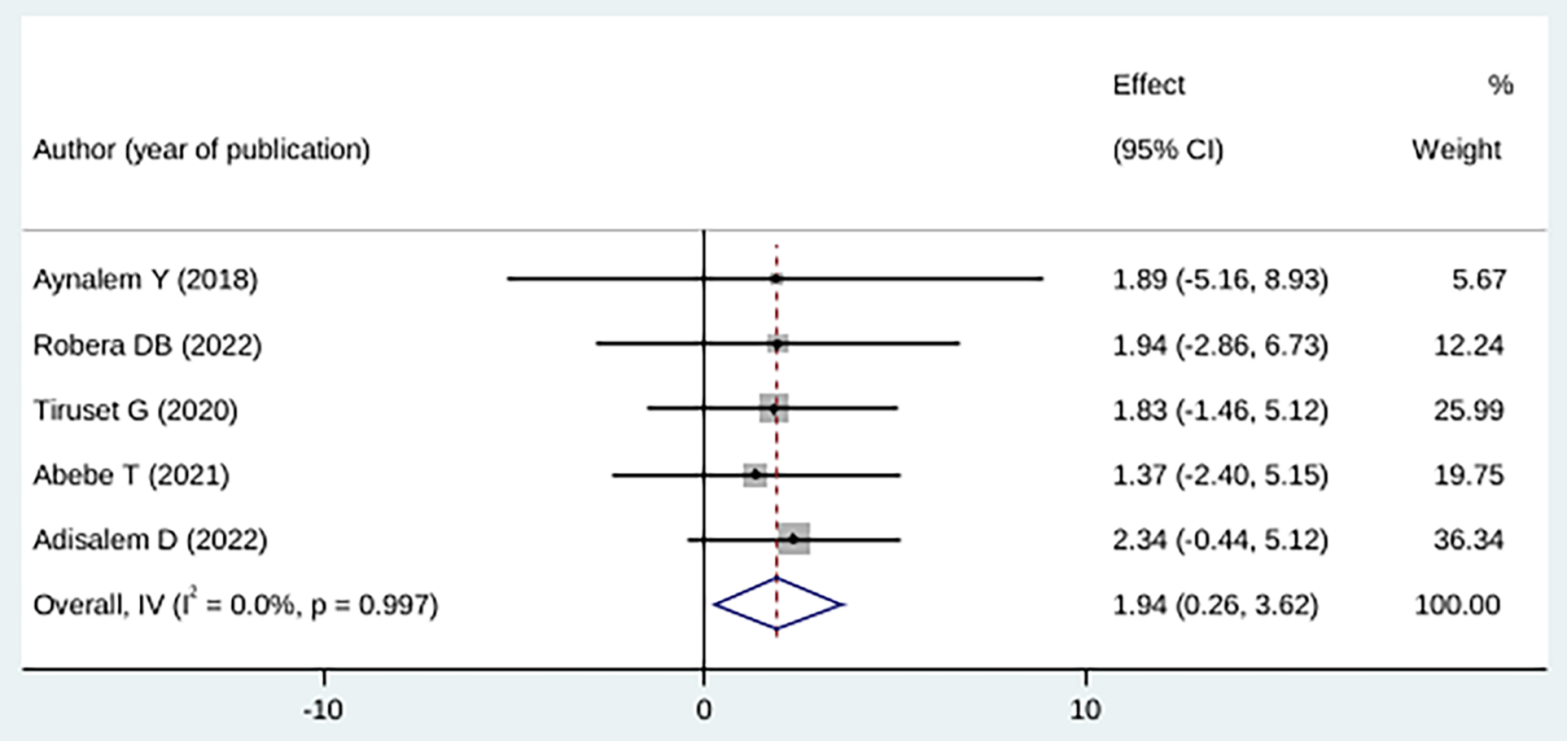
Forest plot showing the association between childbirth-related fear, and pregnancy and obstetrics-related complications.

Women who experienced pregnancy and obstetric complications were 94% more likely to suffer from fear of childbirth, although this finding was not statistically significant (OR: 1.94 (95% CI (0.26, 3.62), *I*^2^: 0.0%. Based on the heterogeneity test (*p* = 0.997), no significant variation was found among the studies. A statistically significant publication bias was not detected by Egger's test (*P* = 0.53) (Figure 6).

#### 3.5.2 Low social support

**Figure 7:**
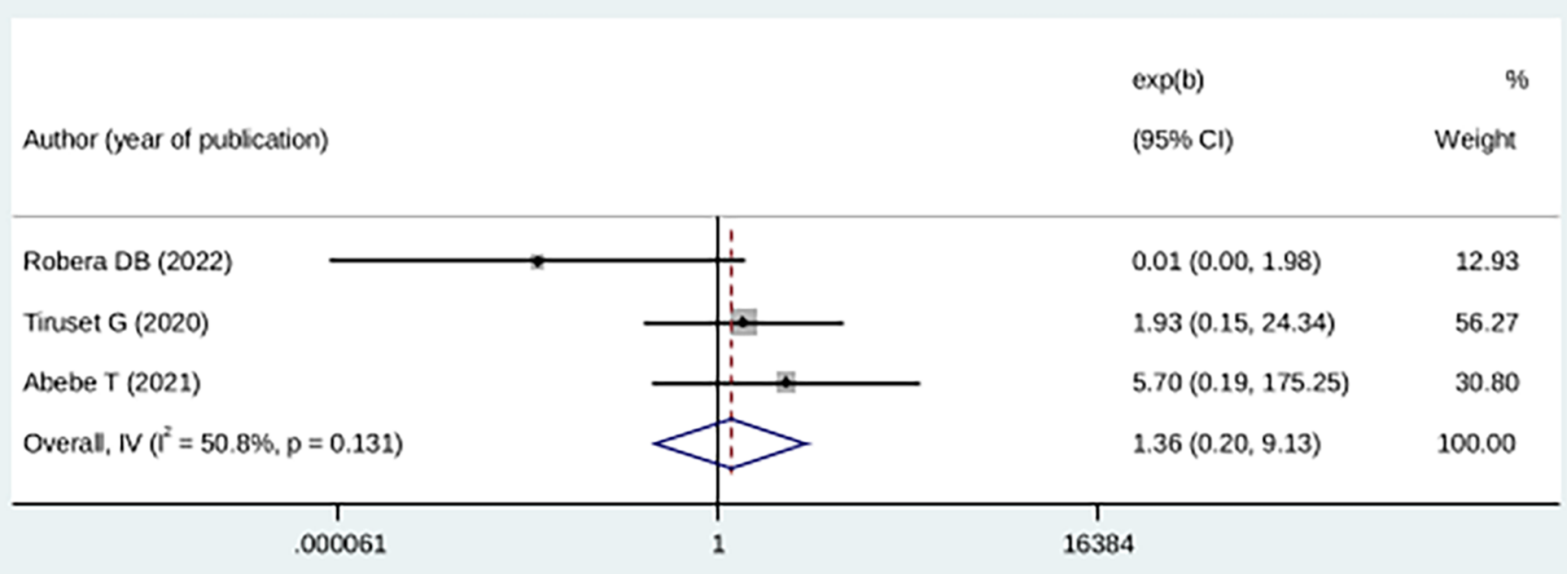
Forest plot showing the association between childbirth-related fear, and lack of social support.

In this study, pregnant mothers with low social support were 36% more likely to develop a fear of childbirth compared with pregnant mothers with high social support; however, the difference was not statistically significant (OR: 1.36 (95% CI (0.20, 9.13), *I*^2^ = 50.8%). As a result of the heterogeneity test (*p* = 0.131), there was no significant difference among the studies. Using Egger's test, we found no statistical significance for publication bias (*P* = 0.44) (Figure 7).

#### 3.5.3 Unplanned pregnancy

**Figure 8:**
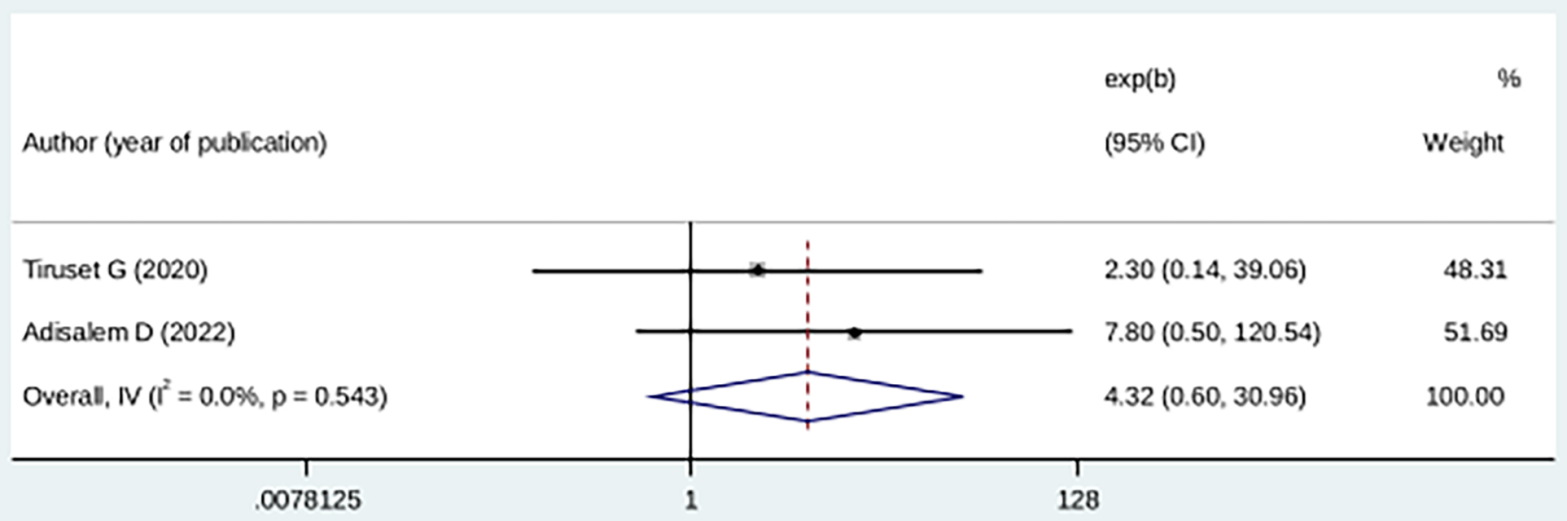
Forest plot showing the association between childbirth-related fear, and unplanned pregnancy.

Compared with pregnant mothers with planned pregnancies, mothers with an unplanned pregnancy history were 32% more likely to experience fear of childbirth, although the difference was not statistically significant (OR: 4.36 (95% CI (0.60, 30.96), *I*^2^ = 0.0). The heterogeneity test did not yield a significant difference among studies (*p* = 0.54) (Figure 8).

#### 3.5.4 Level of education

**Figure 9:**
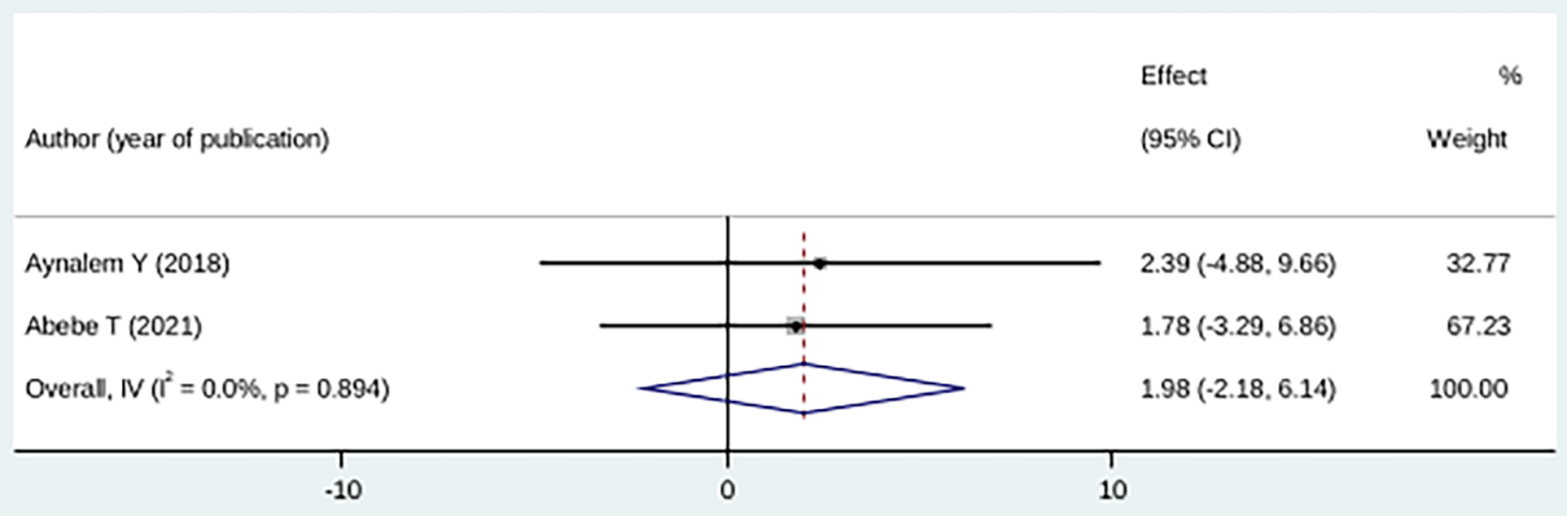
Forest plot showing the association between childbirth-related fear, and no formal education/primary education.

Mothers who had primary and no formal education were 1.98 times more likely to experience fear of childbirth during pregnancy compared with those who had high educational level, although the difference was not statistically significant (OR: 1.98 (95% CI (−2.18, 6.14), *I*^2^ = 0.0). The heterogeneity test did not yield a significant difference among studies (*p* = 0.89) (Figure 9).

## 4 Discussion

This study conducted in Ethiopia assessed the fear of childbirth among pregnant women using a systematic review and meta-analysis approach. We recognized that there was a lack of comprehensive data on childbirth fears in Ethiopia; therefore, we undertook this study to provide a pooled measure of fear of childbirth among pregnant mothers in the country.

The study found that the pooled magnitude of severe fear of childbirth among pregnant mothers in Ethiopia was 21% with a 95% confidence interval of 19–22; *I*^2^ = 0.00%, *P* value <0.001. This prevalence is consistent with similar studies conducted in Malawi, Sweden, and Kenya. In Malawi, a study reported a prevalence of 20% (7) for severe fear of childbirth, whereas in Sweden and Kenya, the prevalence rates were 22% (43) and 22.1% (44) respectively. These findings suggest that the fear of childbirth is a significant concern among pregnant women across different countries, including Ethiopia.

However, this study found a higher prevalence than previous studies. For example, the pooled prevalence in the Globe and Europe was 14% (6) and 11% (45) respectively, in USA 7.7% (46), in Sudan 11.1% (47). In contrast, this study's finding is lower than that found 58.6% in Kenya (8), 82.6% in Turkey (48), 89.3% in Iran (49), 32.4% in Australia (50), 25% in Slovenia (51). One possible reason for the variation in prevalence could be that healthcare providers in some countries, including Ethiopia, may not actively inquire about and address childbirth fears during prenatal care and delivery. This lack of attention to the issue may contribute to the persistence of fears among pregnant women. In addition, variation in the prevalence of fear of childbirth could also be influenced by socioeconomic and cultural norms. Different countries may have distinct societal attitudes and expectations regarding childbirth, which can impact the level of fear experienced by pregnant women. Developing countries, including Ethiopia, may have inadequate services specifically designed to address and prevent the fear of childbirth among pregnant women. Antenatal care programs may not sufficiently cater to the needs of primigravid women in preparing them for childbirth, which may contribute to higher levels of fear. Furthermore, variation in the demographic and maternal characteristics of the study populations across different countries could also contribute to the differences in the prevalence of childbirth. Factors such as education level, access to healthcare, and social support networks can vary among populations and influence fear during childbirth.

The subgroup analysis showed that the prevalence of fear of childbirth in Oromia (25%) was similar to that of the South Nations Nationalities and Peoples' Region (SNNPRs) (24%). The fact that the Oromia and SNNPR regions are spatially adjacent and share a common culture, as well as similar socioeconomic and demographic characteristics, could contribute to the similarity in the prevalence of fear of childbirth between these regions. Cultural norms and socioeconomic factors can influence the perception and experience of childbirth, including the level of fear among pregnant women. The higher prevalence of fear of childbirth in the Oromia and SNNPR regions compared with Addis Ababa could be attributed to the higher percentage of educated women in the capital city. Education plays a crucial role in maternal healthcare use and the uptake of modern health services. Women with higher levels of education tend to have better awareness, knowledge, and access to healthcare, which may contribute to lower levels of fear during childbirth. Moreover, gender inequalities, including limited autonomy for women, may contribute to lower healthcare use and higher levels of fear of childbirth. In regions where women have less control over resources and decision-making processes, they may face barriers to accessing healthcare services and adopting healthy lifestyle options. However, in regions where women are educated and have greater reproductive autonomy, they are more likely to seek and use maternal healthcare services, which can reduce the fear of childbirth. Economic variations can significantly influence antenatal and delivery care. If women do not have control over economic resources, it can impact their ability to access and use healthcare services. Economic constraints may contribute to higher levels of fear of childbirth due to limited access to quality care and support.

In Ethiopia, mothers who had pregnancy- and obstetrics-related complications influenced fear of childbirth, although this was not statistically significant. This result was consistent with those of studies in Tanzania (52), Vietnam (53), and Norway (54). Some possible explanations for their fear include the following: women who have experienced pregnancy and obstetric complications, such as perineal tears, unplanned or emergency cesarean sections, shoulder dystocia, or neonatal mortality, may develop a fear of childbirth during subsequent pregnancies. Negative past experiences can lead to anxiety and fear about the potential recurrence of complications in future pregnancies. Pain experienced during labor has been reported as a significant factor that increases the risk of fear of childbirth in subsequent pregnancies. Intense or traumatic pain during a previous childbirth experience can create a fear of experiencing similar pain in future deliveries (55). Likewise, parous women who have had negative pregnancy and birth experiences may request a cesarean section for their subsequent pregnancies. This preference for cesarean-section delivery can be driven by the desire to avoid repeating negative experiences and associated pain during vaginal childbirth (56). In addition, nulliparous women who have not yet experienced childbirth may also have fear of pain, loss of control, and unknown aspects of the childbirth process. The anticipation of the pain and uncertainty surrounding the childbirth experience can contribute to fear and anxiety in these women.

Pregnant mothers without social support influenced fear of childbirth (FOC), although this was not statistically significant. According to this research, these results are consistent with those reported in China (57), Australia (50), Denmark (58). On the contrary, a study conducted in China revealed that social support has a negative correlation with FOC (57). Social support plays a crucial role in mitigating adverse birth outcomes, pregnancy complications, and childbirth fear. The potential explanations are that mothers benefit greatly from emotional and instrumental support during pregnancy, childbirth, and the postpartum period. Emotional support involves providing empathy, understanding, and a listening ear to help mothers cope with emotional turmoil and challenges. Instrumental support refers to practical assistance, such as help with household tasks, childcare, or transportation, which can alleviate the burden and stress on mothers. Affectionate support involves expressing love, care, and nurturing toward mothers and their infants. This can come from partners, family members, friends, or healthcare providers. Affectionate support helps create a positive and secure environment for both the mother and baby, enhancing the emotional bond and well-being of both. Social support plays a vital role in reducing stress, depression, and anxiety in mothers. Having a network of supportive individuals who can provide encouragement, assistance, and companionship can help alleviate the emotional strain associated with motherhood. Positive interactions with others can provide a sense of belonging, validation, and reassurance.

Despite being not statistically significant, unplanned pregnancy had an impact on fear of childbirth (FOC). This finding is consistent with various studies in China (59), Poland (60), Iran (61). Turkey (62). There is considerable evidence that women with unintended pregnancies experience mental health complications such as perinatal depression, stress, and lower levels of psychological well-being and life satisfaction (63–66). The possible justification is that the pressure of social acceptance, accidentally becoming pregnant, and the lack of choices may leave mothers feeling sad, lonely, anxious, stressed, and guilty. Moreover, the women might also be afraid of getting pregnant again. Likewise, during the antenatal care period, there is no plan made to assist the mothers in creating a successful coping strategy for unintended pregnancy.

The experience of fear of childbirth was more prevalent for mothers with no formal education or only primary education. This result is in line with the findings in Malawi (7), Kenya (44), Sweden (67), and Denmark (58). According to several studies, the utilization of maternal health care (MHC) and maternal education have a beneficial link (68, 69). This might be because uneducated moms are unable to adopt healthy lifestyle choices, which prevents them from using family planning or contraceptive methods to prevent short birth intervals, abortion due to unwanted pregnancy, and pregnancy-related complications. Furthermore, women's education has a positive effect on their cognitive abilities, financial standing, and autonomy, which would increase the demand for health care and lower the hazards that women may experience during and after pregnancy.

## 5 Limitations of this study

Despite being the first systematic review and meta-analysis conducted in Ethiopia, this study has some limitations, such as it could not determine the magnitude of nulliparous and multiparous mothers separately, and our sample size was limited, so we could not detect a significant association between factors and childbirth-related fear.

## 6 Conclusion

The findings of this meta-analysis showed that fear of childbirth is currently more common among pregnant mothers in Ethiopia, and some risk factors are to blame for the exposure, including unplanned pregnancy, lack of social support, no formal or having primary education, and pregnancy and obstetric complications, although not statistically significant. Therefore, it would be better for the Ministry of Health to integrate prevention-based services for mothers with childbirth fears into the antenatal care model. To increase maternal health awareness and knowledge, health education programs need to be strengthened in maternal health care. In the hospital setting, talking therapy, enhanced midwifery care, and alternative interventions could be better used to support mothers.

## Data Availability

The original contributions presented in the study are included in the article/Supplementary Material, further inquiries can be directed to the corresponding author.
